# Evaluation of *Spirulina platensis* as a Feed Additive in Low-Protein Diets of Broilers

**DOI:** 10.3390/ijms26010024

**Published:** 2024-12-24

**Authors:** Hüseyin Yalçınkaya, Sakine Yalçın, Muhammad Shazaib Ramay, Esin Ebru Onbaşılar, Buket Bakır, Fatma Kübra Erbay Elibol, Suzan Yalçın, Awad A. Shehata, Shereen Basiouni

**Affiliations:** 1 Department of Border Control for Animal and Animal Products, Directorate General for Food and Control, Ministry of Agriculture and Forestry, 06510 Ankara, Turkey; huseyin.yalcinkaya@tarimorman.gov.tr; 2 Department of Animal Nutrition and Nutritional Diseases, Faculty of Veterinary Medicine, Ankara University, 06110 Ankara, Turkey; sayalcin@ankara.edu.tr (S.Y.); shazaibramay_7sky@yahoo.com (M.S.R.); 3 Department of Animal Husbandry, Faculty of Veterinary Medicine, Ankara University, 06110 Ankara, Turkey; onbasilar@ankara.edu.tr; 4 Department of Histology and Embryology, Faculty of Veterinary Medicine, Tekirdağ Namık Kemal University, 59030 Tekirdağ, Turkey; buketbakir@nku.edu.tr; 5 Department of Biomedical Engineering, Faculty of Engineering, TOBB Economics and Technology University, 06560 Ankara, Turkey; fatmakubra.erbay@gmail.com; 6 Department of Food Hygiene and Technology, Faculty of Veterinary Medicine, Selçuk University, 42003 Konya, Turkey; syalcin@selcuk.edu.tr; 7 Department of Chemistry, TUM School of Natural Sciences, Bavarian NMR Center (BNMRZ), Structural Membrane Biochemistry, Technical University of Munich, 85748 Garching, Germany; 8 Institute of Molecular Physiology, Johannes-Gutenberg University, 55128 Mainz, Germany

**Keywords:** antioxidant status, broiler, lipid oxidation, low-protein diets, meat quality, performance, *Spirulina platensis*

## Abstract

*Spirulina platensis* is a natural antioxidant product that has the ability to improve the performance of poultry. Therefore, the present study aimed to evaluate the effect of using *Spirulina platensis* as a feed additive in broiler diets. A total of 252 daily male Ross 308 chicks were randomly assigned to six groups. There were two different protein groups: one was at the catalog protein value, and the other was reduced by 10%. *Spirulina platensis* at 0, 0.1, and 0.2% was added to each protein group. The trial lasted 41 days. Reducing the protein level by 10% had a negative impact on the performance of the chicks. However, *Spirulina platensis* supplementation had a positive effect on the feed conversion ratio, reduced the oxidative stress index in the chicks’ liver and meat, increased the total antioxidant status and antioxidant enzyme activities, improved the villus height, serum IgG, and some bone parameters, and reduced the serum triglyceride concentration. The carcass yield, visceral organ weight percentages, total phenolic content, and malondialdehyde (MDA) level in the thigh meat and some serum biochemical parameters were not affected by the usage of *Spirulina platensis*. In conclusion, 0.1% *Spirulina platensis* could be a feasible feed additive in low-protein diets due to eliciting an improved performance, antioxidant status, and immune response in broilers.

## 1. Introduction

There are about 200,000-800,000 species of microalgae, of which only about 35,000 species that have been identified having been commercially produced [1]. Among these, *Spirulina platensis* is particularly rich in proteins, essential amino acids, minerals (especially iron and zinc), essential fatty acids (notably γ-linolenic acid), and carotenoids, which are antioxidant pigments. Spirulina is considered an alternative protein source due to its high protein content (55–70%) and amino acid profile similar to that of soybeans; while the amino acid composition of Spirulina protein can vary, it typically contains methionine, tryptophan, and other amino acids comparable to those found in casein, resulting in a balanced amino acid profile [2]. Several researchers [3,4,5,6,7] have reported that using *Spirulina platensis* in broiler diets improves their body weight, weight gain, and feed conversion ratio (FCR). Abdelfatah et al. [8] concluded that supplementing broiler diets with *Spirulina platensis*, notably 0.5%, can improve their productivity and profitability by promoting weight increase, feed utilization, antioxidant status, immunity, and gastrointestinal health.

In broiler production, rapid growth and high yields are achieved by feeding broilers compound feeds that are high in protein and energy. However, such feeding practices lead to excessive nitrogen excretion, contributing to environmental pollution. In recent years, the reduction of nitrogen excretion has become a focal point in poultry production, particularly from an environmental standpoint. Numerous studies have investigated methods to reduce the protein levels in poultry diets [9,10,11,12,13]. In these studies, the protein levels in compound feeds were reduced in various ways and at different rates. While recent rapid increases in poultry production have shown that lowering protein levels can negatively affect the performance of poultry, broiler feed has been supplemented with amino acids, feed additives, and feed ingredients to avoid such negative impacts. In addition, broilers fed low-nutrient-density diets were found to have a reduced total antioxidant capacity and diminished superoxide dismutase (SOD) activity in the jejunal mucosa [13].

Spirulina contains salicylic, trans-cinnamic, synaptic, chlorogenic, quinic, and caffeic acids, which are responsible for antioxidant activity individually or synergistically [14]. Dietary spirulina supplementation positively affected the health and performance of broilers [3,6,15]. Bonos et al. reported that dietary *Spirulina platensis* supplementation affected the fatty acid composition of broiler meat without any significant negative impact on performance parameters or the meat oxidative stability [16]. In another study, an increase in beneficial omega-3 polyunsaturated fatty acids (n-3 PUFAs) and a reduction in lipid oxidation were seen with increased *Spirulina platensis* intake [17]. *Spirulina platensis*, rich in bioactive compounds such as phycocyanin, carotenoids, and essential amino acids, possesses antioxidant properties that help mitigate the effects of low-protein diets by boosting the activity of key antioxidant enzymes, including SOD and glutathione peroxidase (GPx) [5]. Furthermore, Alagbe et al. [15] observed that dietary Spirulina supplementation significantly enhanced the serum catalase (CAT) levels, total antioxidant capacity, ileal villus perimeter, tibia bone ash, and relative mRNA expression of heme oxygenase 1, SOD, claudin 1, and tumor necrosis factor-alpha in the jejunal mucosa of birds on day 26 [15]. *Spirulina platensis* also positively affects bone growth and biomechanical bone features [18].

The potential of *Spirulina platensis* as a partial replacement for traditional protein sources in broiler diets has been explored [19]; however, its effects in protein-reduced diets have not been thoroughly investigated. Therefore, it is necessary to clarify the effects of *Spirulina platensis* on antioxidant metabolism in broilers. Additionally, there is a need for studies on the practical application of lowering protein levels in compound feeds while preventing performance losses by supplementing diets with *Spirulina platensis*, which is rich in bioactive compounds and possesses potent antioxidant properties. Therefore, the present study aimed to determine the effects of using different levels of *Spirulina platensis* as a feed additive in low-protein diets on the performance, carcass characteristics, antioxidant status, meat quality, intestinal histomorphology, bone quality, and some blood biochemical indices of broilers.

## 2. Results

**Figure 1:**
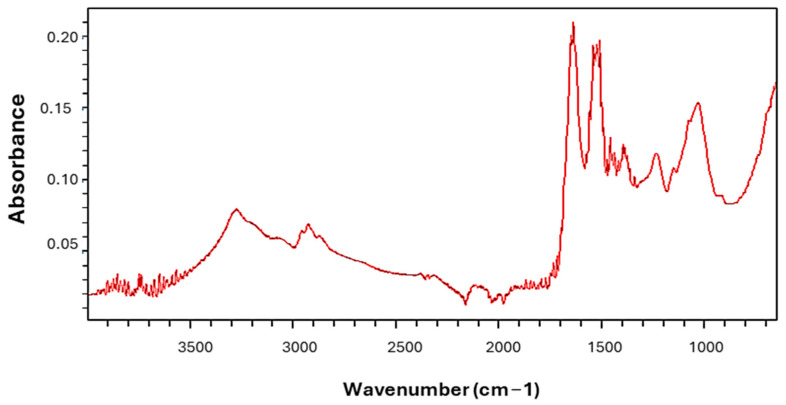
Fourier transform infrared spectrum of *Spirulina platensis.*

*Spirulina platensis* has 69.73% crude protein and 3.36% ether extract, as shown in Table 1. It was mainly rich in total glutamic acid (7.035%). The palmitic acid, gamma-linolenic acid (GLA), and linoleic acid levels were 43.76, 22.39, and 13.76% of the total methyl esters of fatty acids, respectively. The oxidative stress index (OSI) value was found to be 0.997%. The graph from the Fourier transform infrared (FT-IR) spectroscopy analysis shows that *Spirulina platensis* has a high number of peaks, showing functional groups, as shown in Figure 1. There was 1 primary compound, 12 minor compounds, and 1 trace compound; a total of 14 compounds were observed in *Spirulina platensis*.

Reducing the crude protein content of the compound feed by 10% from the catalog values significantly decreased (*p* < 0.001) the means of the body weights of the broilers on days 10 and 24, and at the end of the 41-day trial (Table 2). Reducing the crude protein content by 10% negatively affected the means of body weight gain during all trial phases (*p* < 0.001, Table 2). Adding *Spirulina platensis* to the compound feeds did not affect the means of body weight and weight gain. No interaction between *Spirulina platensis* and protein levels was observed in terms of body weight or body weight gain.

Reducing the crude protein content in compound feeds by 10% significantly reduced the mean feed intake (*p* < 0.05, Table 3). No effect of Spirulina supplementation was observed in the groups fed diets containing both protein levels. The addition of *Spirulina platensis* to broiler compound feeds positively affected FCR values during the periods of 25–41 and 0–41 day (*p* < 0.05, Table 3). Reducing the crude protein level significantly increased (*p* < 0.001) the feed intake per kg of body weight gain from day 11 to the end of the trial. No interaction was observed between the Spirulina level and crude protein level in terms of the FCR. Reducing the crude protein level decreased the European Production Efficiency Factor (EPEF) (*p* < 0.001). No differences were observed in the EPEF values with the addition of *Spirulina platensis* at 0, 0.1, and 0.2% levels to both normal and low crude protein compound feeds (Table 3).

Throughout the trial, one mortality was observed in both 0.2% Spirulina-supplemented groups. The livability percentages for the groups fed diets supplemented with 0, 0.1, and 0.2% *Spirulina platensis* were 100%, 100%, and 97.62%, respectively, for both protein groups at each level.

Reducing the crude protein contents of compound feeds by 10% resulted in a decrease in the carcass yield (*p* = 0.020) and an increase in the liver and abdominal fat percentages (*p* < 0.01, Table 4). The addition of *Spirulina platensis* at different levels did not affect the carcass yield or the weight of certain internal organs.

The L* value in the breast meat and the b* value in the thigh meat increased with the addition of *Spirulina platensis* (*p* < 0.05, Table 5). The b* value in the thigh meat also increased as a result of the reduction in the crude protein content (*p* = 0.042). An interaction between the *Spirulina platensis* level and crude protein level was also observed in the L* value of the breast meat (*p* = 0.031). No effect of different levels of *Spirulina platensis* added to the compound feeds containing both normal and low protein levels was observed on the total phenolic content or MDA levels in the thigh meat of the broilers (Table 5).

*Spirulina platensis* supplementation reduced the value of OSI (*p* < 0.001, Table 6). The addition of *Spirulina platensis* significantly increased the activities of CAT (*p* = 0.013), SOD (*p* < 0.001), and GPx (*p* = 0.011) in the liver and the GPx enzyme activity (*p* = 0.037) in the breast meat. No effects of the protein level on the antioxidant–oxidant status or antioxidant enzyme levels in liver and breast meat were observed, nor were any interactions between *Spirulina platensis* and the protein levels.

There were no significant effects of different supplementation levels of *Spirulina platensis* in the diets containing both normal and low levels of crude protein on the fatty acids and methyl esters in the breast meat, as shown in Table 7. The ratio of omega 6 fatty acids to omega 3 fatty acids and the percentages of stearic acid, arachidic acid, gamma-linolenic acid, and eicosapentaenoic acid were not affected by the dietary protein level. However, the dietary protein levels were shown to significantly affect the levels of other fatty acids. The total mono-unsaturated fatty acids (MUFA), polyunsaturated fatty acids (PUFA), and unsaturated fatty acids (UFA) were significantly affected by the dietary protein levels. An interaction between the *Spirulina platensis* level and crude protein level was observed in the percentages of palmitoleic acid and linoleic acid, total MUFA and PUFA.

*Spirulina platensis* supplementation increased the villus height (VH) and crypt depth (CD) and significantly decreased the ratio of the VH to the CD in the duodenum (*p* < 0.001), as shown in Table 8. However, Spirulina increased the VH and the ratio of the VH to CD (*p* < 0.005) in the jejunum and ileum. When the protein level was reduced by 10%, the villus height was decreased, and the ratio of the VH to the CD was increased in the duodenum and jejunum (*p* < 0.01). The VH and the ratio of the VH to the CD in the ileum were reduced by reducing the protein levels. No significant interactions between the *Spirulina platensis* level and the crude protein level were found in the ratio of the VH/CD in the jejunum or in the CD in the ileum.

Dietary *Spirulina platensis* addition increased the displacement at yield load in the tibia and the ultimate load in the femur (*p* < 0.01), as shown in Table 9. Other bone properties were not affected by Spirulina supplementation. The dietary protein levels affected the tibia properties (*p* < 0.01). However, only the YL in the femur was affected significantly by the dietary protein level. No significant interactions between the *Spirulina platensis* and crude protein levels were observed in the tibia or femur properties.

With the addition of *Spirulina platensis* to compound feeds, the serum triglyceride levels decreased (*p* = 0.049) and the IgG increased (*p* < 0.001), while reducing the protein level resulted in an increase in the triglyceride level (*p* = 0.002) and a reduction in the IgG level (*p* = 0.001), as reported in Table 10. There were no significant differences in the serum total protein, cholesterol, alkaline phosphatase (ALP), aspartate aminotransferase (AST), or alanine aminotransferase (ALT) levels among the groups. No significant interactions between the *Spirulina platensis* level and crude protein level were found in the blood biochemical parameters.

## 3. Discussion

The crude protein content of the *Spirulina platensis* used in this study (69.73%) is higher than the crude protein levels reported by some researchers [20,21]. The crude protein (70.7%) found by Kumar et al. [22] is similar to that obtained in this study. Habib et al. [23] reported the crude protein content of *Spirulina platensis* to be between 55% and 70%, depending on the source. Oguzkan et al. [24] found the Total antioxidant status (TAS) value of *Spirulina platensis* to be 4.142 mmol Trolox equivalent/L and reported a TAS reference value above 2 as favorable. In the present study, the TAS value of the *Spirulina platensis* used was 2.36 mmol Trolox equivalent/kg, above 2. This indicates that the *Spirulina platensis* used in this study has a relatively high antioxidant capacity.

The volatile oil compounds found in the *Spirulina platensis* comprised 1 primary compound (>5%), 12 minor compounds (1–5%), and 1 trace compound (<1%), according to their presence in the volatile oils classification reported by Kılınç et al. [25]. The major compounds are n-tetradecane and n-alkanes, which have antioxidant, antimicrobial, flavor, and hypocholesterolemic effects [26]. One of the minor compounds, 5,6,7,7A-tetrahydro-4,4,7A-trimethyl-2(4H)-benzofuranone, which is a triterpene, has antimicrobial properties. Alkanes, alkenes, aldehydes, aromatics, carboxylic acids, esters, ketones, alcohols, phenols, amides, amines, imines, and oximes were detected with functional groups of C-H, C=C, C-O, C=O, C-N, C=N, O-H, and N-H, as reported by Kılınç et al. based on absorption peaks being observed at the corresponding wavelengths [25]. The peak around 3290 cm^−1^ could be attributed to the CH stretching group [27]. The absorption peaks located at 2970–2860 cm^−1^ may be due to the presence of C-H stretching vibrations [28,29]. The peaks in the region of 2390–2320 cm^−1^ could be attributed to O-C-O, C=N, C=O, and N=C=S stretching [27]. The peaks at 2120–1230 cm^−1^ could be assigned to the C=O, C=C, C=S, C-N, C=N, and N-O, C-O stretches of proteins, acid halides, aldehydes, ketones, sulfur compounds, amino acids, amines, nitro compounds, and aromatic esters [27,28,30]. The peaks in the region of 1240–900 cm^−1^ could be attributed to the C=O, C-O, C-C-O, C-O-H, C=C, N-H, and O-H stretching of carboxylates, alcohols, alkenes, proteins, and phenolic groups [27,30]. In the study of Dotto et al. [31], major intense bands were observed around 3269, 2918, 1660, 1627, 1548, 1409, 1028, 850, 700, and 543 cm^−1^. Çelekli et al. [28] reported major intense bands around 3265, 2908, 1642, 1396, and 1024 cm^−1^. The differences among peaks may be due to the manufacturing processes of *Spirulina platensis*.

In the present study, reducing the protein content in diets had a statistically significant negative effect on the live weight gain, feed consumption, and feed efficiency. It has been reported that reducing the crude protein content in starter diets from 22.2% to 16.2% [9], from 22% to 16% [32], and from 24% to 18% [33] negatively affected the body weight, weight gain, and FCR. Furthermore, Awad et al. [9] reported that feed consumption decreased, and mortality increased with a reduction in dietary protein. Similar to the findings of this research, Yuan et al. [33] also noted that the survival rate of broilers was not affected by their dietary protein levels. Ribeiro et al. [34] observed a significant interaction between dietary protein levels and algal extract supplementation in terms of body weight, weight gain, feed consumption, and FCR when the dietary protein was reduced from 21% to 17% in 21-day-old broiler chicks. However, no significant interaction was found between dietary protein levels and *Spirulina platensis* supplementation in terms of body weight, weight gain, feed consumption, and FCR in the present study.

Some researchers [3,4,5,6,7] have reported that the inclusion of *Spirulina platensis* in the diets of broilers improved their body weight, weight gain, and FCR. Raju et al. [35] reported that adding Spirulina at a level of 0.05% to feed contaminated with 300 ppm of aflatoxin mitigated the negative effects of aflatoxin on growth performance. Park et al. [5], in a 35-day trial, observed a linear positive effect on weight gain, the FCR, and the EPEF with the addition of *Spirulina platensis* at levels of 0.25%, 0.50%, 0.75%, and 1.0%, but the feed consumption and mortality were not affected by the supplementation. The positive effect on the performance and EPEF was attributed to the chemical composition and physiological functions of *Spirulina* [5]. Spirulina possesses potential antimicrobial, antioxidant, and anti-inflammatory biological properties, as well as immune-enhancing effects due to its carotenoid pigments, phycocyanin, polyunsaturated fatty acids, vitamins, macro- and micro-elements, and other chemical compounds [5,36]. The positive effect on performance observed in this study may be attributed to the potential antioxidant properties of *Spirulina platensis*.

Mullenix et al. [37] found that supplementing low-protein diets (17%) with 10% *Spirulina platensis* impaired FCR. This was due to the limiting amino acids in low-protein diets that mask any effect of Spirulina [37]. The variations in the results observed in the studies regarding the use of *Spirulina platensis* may be due to factors such as the composition of *Spirulina platensis*, the bioactive compound content, the dosage used in the diets, the trial duration, and the dietary composition used.

In this study, reducing the crude protein content of compound feeds by 10% decreased the carcass yield and increased the liver and abdominal fat percentages. The addition of *Spirulina platensis* at different levels did not affect the carcass yield or the weight of certain internal organs. Similar to the findings of this study, other researchers have reported that reducing dietary crude protein levels increased the relative weight percentages of the liver [32,38] and abdominal fat [39,40]. The increase in relative liver weight has been attributed to enhance de novo lipogenesis [41]. The reduction in the abdominal fat relative weight in response to a low-protein diet has been linked to an increase in the energy/protein ratio of the diet [32,42]. In the present study, reducing the dietary crude protein by 10% increased the energy/protein ratio by 11.11%. Some researchers [9,33] did not observe any differences in the relative weight percentages of liver when the dietary protein levels were reduced. Awad et al. [9] attributed the lack of difference in the abdominal fat weight percentage to the young age (21 days) of the broilers fed low-protein diets.

Some researchers observed that Spirulina supplementation increased dressing percentage [4,8,43,44], and relative weight of the liver [43], decreased the relative weight of abdominal fat [45,46], and did not affect the relative weights of abdominal fat (5, 43, 44), gizzard [5,8,43,47], heart [8,43,47] and liver [47].

In the current study, the supplementation of the *Spirulina platensis* at different levels to the compound feeds containing normal and low protein levels increased the L* value in the breast meat and the b* value in the thigh meat. The b* value in thigh meat also increased with the reduction of crude protein content. An interaction between *Spirulina platensis* level and crude protein level was observed in the L* value of breast meat. A linear increase in the L* value of breast meat was observed with increasing levels of *Spirulina platensis* in feeds containing normal protein levels. Park et al. [5] reported that color parameters (L*, a*, b*) in broiler meat were not affected by the addition of different levels of *Spirulina platensis* during a 35-day trial. However, higher levels of *Spirulina platensis* influenced the yellow and red colors of the meat. The addition of Spirulina at a level of 10% increased redness (a*) and yellowness (b*) in breast meat, thigh meat, and skin [37]. Toyomizu et al. [48] found that the supplementation of 4% Spirulina increased redness in breast meat, while an 8% level did not, and Spirulina increased the yellowness of breast meat. Pestana et al. [49] also noted an increase in yellowness in both breast and thigh meat with Spirulina supplementation. Raach-Moujahed et al. [36] reported that yellowness in the skin and breast meat increased by adding 2.5% Spirulina to the diet, while no such effect was observed with a 5% addition. Venkataraman et al. [50] found that replacing peanut meal and fishmeal with *Spirulina* at 17% and 14% caused darker coloration in the skin, breast, and thigh muscles. The increase in pigmentation is attributed to the pigment compounds in Spirulina. Spirulina contains zeaxanthin (0.1–0.7 mg/g), total carotenoids (0.28–2.23 mg/g), and C-phycocyanin (1.1–9.1 mg/g) [5]. Zeaxanthin accumulation in breast meat is directly associated with the yellowness observed in the breast meat and is the primary carotenoid pigment in Spirulina [48]. The differences in meat color observed in the studies may be due to the variations in trial design, dose used in the study, genetic differences, and differences in the chemical composition of Spirulina [37]. In line with the findings of the literature, the increase in thigh meat yellowness (b*) in the present study can be attributed to the total carotenoid and total phycocyanin content of the *Spirulina platensis* used.

In the present study, no significant effects were observed on the total phenolic compounds and MDA levels in thigh meat from broilers fed diets containing different levels of crude protein and supplemented with varying doses of *Spirulina platensis*. Sutton et al. [51] reported that broilers fed with low protein had a decreased plasma SOD activity and lipid peroxidation compared with normal protein under thermoneutral conditions. In the present study, the dose of *Spirulina platensis* added to each protein group did not affect the levels of total phenolic compounds in thigh meat. In agreement with these findings, Pestana et al. [49] also reported that supplementation of *Spirulina platensis* did not affect MDA levels in breast and thigh meat. *Spirulina platensis* enhances antioxidant activity in poultry due to its rich C-phycocyanin, an antioxidant biliprotein pigment with hypolipidemic activity [52,53]. The increase in TAS levels and antioxidant enzyme activities observed in the present study supports this hypothesis. However, Pestana et al. [49] did not observe an increase in antioxidant capacity with 15% Spirulina supplementation. Park et al. [5] reported linear increases in serum antioxidant enzyme activities (SOD and GPx) with increasing levels of Spirulina in the diet. This increase may also be attributed to phycocyanin, β-carotene, and phenolic compounds present in *Spirulina* [5]. In the present study, no significant effects were observed on the total phenolic compounds and MDA levels in thigh meat from broilers fed diets containing different levels of crude protein and supplemented with varying doses of *Spirulina platensis*. Sutton et al. [51] reported that broilers fed with low-protein diets had a decreased plasma SOD activity and lipid peroxidation compared with those fed diets with normal protein levels under thermoneutral conditions. In the present study, the dose of *Spirulina platensis* added to each protein group did not affect the levels of total phenolic compounds in the thigh meat. In agreement with these findings, Pestana et al. [49] did not observe an increase in antioxidant capacity with 15% Spirulina supplementation. Park et al. [5] reported linear increases in serum antioxidant enzyme activities (SOD and GPx) with increasing levels of Spirulina in the diet. This increase may also be attributed to phycocyanin, β-carotene, and phenolic compounds present in *Spirulina* [5]. GPx and SOD enzymatically act as free radical scavengers within cells [54]. Estrada et al. [55] reported that Spirulina protein extract scavenges on hydroxyl radicals, with phycocyanin being the primary component responsible for its antioxidant activity. Additionally, β-carotene and other carotenoids in Spirulina protect cells from oxidative stress by preventing oxygen damage through various mechanisms [56]. Abdelfatah [8] also reported that TAC, CAT, GPx, and SOD levels in serum were higher in the Spirulina groups than those in the control group.

Our study found that varying doses of *Spirulina platensis* supplementation in broiler diets with different crude protein levels did not significantly affect the meat’s total phenolic compounds or MDA content. However, there was an increase in the TAS levels and antioxidant enzyme activity. Despite the absence of significant oxidative stress markers, the enhanced activities of CAT, SOD, and GPx suggest an improved antioxidant response, particularly in the liver. This increase in enzyme activity may indicate that the liver was exposed to elevated oxidative stress, which triggered these enzymes in order to counteract reactive oxygen species [57,58]. Additionally, the muscle tissue responded by increasing its GPx activity alone, suggesting a tissue-specific effect of Spirulina supplementation. Further studies are needed to investigate the underlying mechanisms and long-term effects of Spirulina supplementation on the oxidative stress and antioxidant defense in different tissues.

Fatty acids are essential substances that make up fats and tissue cells, which determine the physicochemical properties of fats and influence the flavor of meat. The fatty acid composition of chicken meat can be regulated by the diet nutritional level of the chicken [59,60]. In the current study, the total SFA and MUFA were increased, and the PUFA and USA were decreased when the dietary protein levels were decreased (*p* < 0.001). The dietary supplementation of *Spirulina platensis* did not affect the fatty acids methyl esters in the breast meat in the present study. Bonos et al. [16] reported that *Spirulina platensis* only affected the lauric acid and trans-linoleic acid levels in broiler breast meat. According to the study of Spinola et al. [17], the fatty acid profile, particularly that of n-3 PUFAs like 18:3n-3, 20:5n-3, and 22:6n-3, shows changes with varying levels of dietary Spirulina. In the present study, the interactions between the *Spirulina platensis* level and protein level were seen in the percentages of palmitoleic acid and linoleic acid, the total MUFA, and PUFA. In the control and Spirulina platensis groups, the percentages of linoleic acid and total MUFA increased, and the PUFA decreased with reduced dietary protein levels. The differences in the results obtained by different studies may be due to the manufacturing processes of *Spirulina platensis*, the doses of Spirulina used, and the ingredients and compositions of diets.

The villus height has a critical role in the intestinal absorption of nutrients. *Spirulina platensis* supplementation increased the villus height and the ratio of the villus height to the crypt depth (*p* < 0.005) in the jejunum and ileum of broilers in the present study, similar to the study of Ansari et al. [61]. Khan et al. [44] also reported that *Spirulina platensis* supplementation increased the intestinal villus height. The increase in the villus height causes an increase in the total absorptive area of the luminal villus, causes an increase in digestive enzyme action, and improves the transport of nutrients to the villus surface in the final stage of the absorption of nutrients [62,63], and this result reflects improvement in the FCR and EPEF. Reductions in the protein level impaired the villus height in all parts of the intestine, which reflects negative effects on the performance characteristics of the broilers. In the control and *Spirulina*-fed groups, the ratio of the villus height over the crypt depth decreased in the duodenum and ileum when the dietary protein levels were reduced.

Dietary *Spirulina platensis* increased the displacement at yield load in the tibia and the ultimate load in the femur (*p* < 0.01). No significant interactions between the *Spirulina platensis* and crude protein levels were observed in tibia or femur properties. Suzer et al. [18] reported that the maximum load (*p* = 0.030), breaking displacement (*p* = 0.015), yield load (*p* = 0.049), post-yield displacement (*p* = 0.028), and work of fracture (*p* = 0.031) in the tibias of rats were significantly higher in Spirulina-fed groups compared to the control group, while the stiffness had a non-significant increase. The positive effects seen in bone characteristics may be the results of the vitamin C, phycocyanin, or collagen properties of *Spirulina platensis*. *Spirulina platensis* may have increased the collagen content of the bones by stimulating vitamin C metabolism [18].

The addition of *Spirulina platensis* to the compound feed lowered the serum triglyceride levels and improved the levels of IgG, whereas reducing the protein level led to an increase in triglyceride and a reduction in the IgG level. Similarly, Qiu et al. [40] stated that the immune function was suppressed in broilers given a low-protein diet. Awad et al. [9] reported that reducing the dietary protein of broilers from 22.2% to 16.2% significantly increased the serum triglyceride levels and did not affect the total protein levels. The elevated serum triglyceride levels observed in the low-protein group can be attributed to a higher energy-to-protein ratio, which enhances the lipogenic activity in the liver [64]. This observation is consistent with the findings of the present study. Similar to the present study, Abdel hak El Shabrawy et al. [65] reported that *Spirulina platensis* extract improved the serum levels of IgG. Ribeiro et al. [34] found no differences in the serum total cholesterol, total lipid, total protein, ALT, AST, or ALP when the dietary protein was reduced from 21% to 17%. However, adding an algal extract to the diet reduced the total cholesterol, HDL, and total lipid levels without affecting the serum total protein, ALT, AST, or ALP levels. Similar to the present study, they reported no interaction between the dietary protein level and the amount of algal extract in terms of serum parameters [33]. However, Qiu et al. [40] found that reducing the dietary crude protein contents decreased the ALP activity.

Shanmugapriya and Babu [6], in a 36-day study, reported that adding *Spirulina platensis* to commercial broiler compound feeds at levels of 0.5%, 1.0%, and 1.5% improved blood parameters. Pestana et al. [49] observed no differences in the serum protein or ALT levels with Spirulina supplementation but noted an increase in the serum lipid and cholesterol levels and a decrease in AST and ALP activity. Variations in the above studies regarding dietary crude protein are likely due to differences in protein levels, the added amino acid content [12], energy levels [66], and factors such as the broiler sex [67] and age [9]. Differences in the effects of *Spirulina platensis* may be attributed to its composition, bioactive compounds, dosage, trial duration, and dietary composition.

## 4. Materials and Methods

### 4.1. Experimental Design and Diets

The present study was approved by the Animal Care and Use Committee of Ankara University, Turkey (2020-9-77). A total of 252 daily Ross 308 male broiler chicks were allocated into six groups, each containing 42 chicks. There were two protein groups: one protein group diet was a normal (catalog) protein diet, and the other was 10% lower than the standard value. For the normal protein diet groups, the requirement levels reported in the Ross 308 catalog were prepared: a starter diet of 23% CP and 3000 kcal/kg ME for 0–10 days of age; a grower diet of 21.5% CP and 3100 kcal/kg for 11–24 days of age; and a finisher diet of 19.5% CP and 3200 kcal/kg ME for 25–41 days of age. Low protein content diets were prepared by decreasing the crude protein levels reported in the Ross catalog by 10% [68]. No changes were made in other nutrient levels. Ingredients and composition of the basal and low-protein diets are given in Table 11.

*Spirulina platensis* at 0, 0.1, and 0.2% was added to each protein group diet. The trial lasted 41 days. Each group was divided into six replicates, with each replicate consisting of 7 chicks. Each replicate was placed in separate pens (90 × 80 cm). Wood shavings were used as litter in each pen. Feed and water were provided for ad libitum consumption, and the diets were presented in mash form. A 23 h light and 1 h dark cycle was applied in the first week of the trial. After the first week, the dark period was gradually increased to 6 h within 3 days, and the 18 h of light and 6 h of dark cycle was applied until the 41st day.

### 4.2. Analysis of Spirulina Platensis and Diets

The nutrient compositions of *Spirulina platensis* and the diets were determined according to the methods of AOAC [69]. Metabolizable energy values were estimated using the equation of Carpenter and Clegg, as reported by Yalçın et al. [70]. For the determination of fatty acid methyl esters (FAME), fatty acids from *Spirulina platensis* were methylated [71] and the obtained fatty acid methyl esters (FAMEs) were analyzed by gas chromatography (HP 6890, Agilent, USA) using an HP-88 column (100 mm × 250 µm × 0.25 µm) (Agilent, USA). To determine the volatile oils profile, GC-MS (Agilent: 6890 MS:5973, Flanders, NJ, USA) using an HP-5 MS column (30 m) was used. Component mass spectra were identified by comparing the retention indices of the components defined in the Flavor2, HPCH1607, and W8N05ST libraries. FT-IR spectroscopy (Agilent Technologies, Cary 630, Petaling Jaya, Malaysia) was used for spectral scan analysis at wavenumbers ranging from 650 to 4000 cm^−1^ to evaluate functional groups that might be involved in the sorption process. TAS (mmol Trolox equivalent/kg) and total oxidant (TOS, mmol H_2_O_2_ equivalent/kg) levels were analyzed using commercial kits (Rel Assay Diagnostics, Gaziantep, Turkey, Cat No: RL0017 and RL0024, respectively) by colorimetric methods. OSI values were calculated as described by Ramay and Yalçın [72].

### 4.3. Performance Parameters

The live weights of the birds were determined by weighing them individually at the beginning of the study (day 0), at the times when the feed was changed (days 10 and 24), and at the end of the trial (day 41). Weight gains were calculated from the differences between the weights. Feed consumption was determined as a subgroup. FCR was calculated as kilograms of feed consumed per kilogram of weight gain. Birds were monitored daily. Livability and EPEF values were calculated according to Onbaşılar et al. [73]. Performance parameters were evaluated on a pen basis.

### 4.4. Carcass, Organ and Meat Parameters

On the 41st day of the experiment, 12 broilers from each group (2 from each pen) were weighed and slaughtered by severing the jugular vein. Hot carcass, liver, gizzard, spleen, heart, and abdominal fat were weighed to determine the carcass yield and relative weights of internal organs. For analysis of antioxidant–oxidant status and some antioxidant enzyme activities, liver samples were taken and stored at −20 °C for 2 days.

Each carcass’s breast meat and leg samples (right and left) were appropriately sliced. The left parts of the thigh meat samples were divided into 3 equal cuts (upper, middle, and lower). Left thigh meat samples were stored at 4 °C for 24 h and used for the thiobarbituric acid reactive substances (TBARS) analysis. Left breast samples were divided into 3 equal cuts (upper, middle, and lower). Upper-left samples were used for fatty acid analyses and stored at −20 °C, and the right parts were stored in the same way for further analysis. The right parts of breast and thigh meat samples after 24 h storage at 4 °C were subjected to color measurements and lipid oxidation analysis. Meat color according to the L* (brightness), a* (redness), and b* (yellowness) coordinate system was measured using Minolta CR400 (Konica Minolta, Tokyo, Japan). Color readings were made from 3 different points on the meat samples.

Total phenolic content analysis in the right part of thigh meat was carried out spectrophotometrically according to the Folin–Ciocalteu method after extraction with water and chloroform [74,75]. Lipid oxidation in thigh meats (after one-day storage at 4 °C) was determined spectrophotometrically by the TBARS method [76], and MDA concentration in thigh meat samples was expressed as mg/kg wet meat. TAS, TOS, and OSI parameters and antioxidant enzyme parameters (CAT (Rel Assay Diagnostics, Gaziantep, Turkey), SOD (Otto Scientific, Otto3047, Nuremberg, Germany)) were determined for liver and breast meat samples according to the manufacturer’s protocol. In addition, GPx was analyzed with the method reported by Paglia and Valentine, for liver and breast meat samples [77].

The fatty acid profiles of breast meat samples were determined using gas chromatography (Shimadzu GC-2010, Shimadzu Co., Kyoto, Japan), and chromatography was equipped with a flame-ionized detector (FID). Total lipids were extracted from 5 g thawed meat samples using the Soxhlet apparatus with diethyl ether [69], and FAMEs were prepared [78]. FAMEs were then separated with a Teknokroma capillary column (TR-CN100, 100 m × 0.25 mm × 0.20 µm). Injection volume was set to 1 µL with a split ratio of 1:100. Helium was used as carrier gas (1 mL/min). The detector gas flows were hydrogen at 30 mL/min, makeup (Helium) at 30 mL/min, and air at 300 mL/min. Both injector and FID temperatures were fixed at 240 °C. The temperature program was as follows: the initial temperature was held at 40 °C for 2 min, then increased to 165 °C for 5 min, 215 °C for 8 min, and finally 240 °C for 8 min at 4 °C/min each. The FAMEs were determined by comparing the retention times and area percentages with a standard FAME mixture (Supelco 37 Component FAME Mix, 1 mL, CRM47885 Supelco).

### 4.5. Intestinal Histology Parameters

Intestinal segments (duodenum, jejunum, ileum) were prepared for morphological measurements, as shown by Onbaşılar et al. [79]. The tissues were washed twice in 0.1 M phosphate buffer solution. After washing, the tissues were fixed on 3% glutaraldehyde for 24 h. Then, the tissue samples were dehydrated through a graduated-acetone series (25, 50, 70, and 100%). Two samples from each part of the intestine (inner and vertical surface) were removed and placed on stubs. FEI brand ‘Quanta FEG 250’ model scanning electron microscope, with technology that does not require vacuum, critical drying, or coating with gold, was used. Thus, direct images were taken from identified tissues, and VH and CD were recorded. The ratio of VH over CD (VH/CD) of each sample was calculated [80].

### 4.6. Bone Parameters

Right leg samples were taken and cleared of all tissues to collect the tibia and femur, which were stored at −18 °C until mechanical testing. For bone strength determination, samples were subjected to three-point bending with material testing machines using Instron Plus software (Instron 5944 testing frame, Instron, Norwood, MA, USA) and a standard 2 kN load cell with a 5 mm/min rate. The span length between the two fixed points supporting the bone was 60 mm for the tibia and 40 mm for the femur. The bones were subjected to load from the mid-shaft until a failure occurred. Load and displacement values were obtained from the testing device during the test and used to acquire a load–displacement graph. Stiffness (N/mm), ultimate load (N), displacement at ultimate load (mm), yield load (N), and displacement at yield load (mm) values were calculated from the raw data. Stiffness values were found by calculating the slope of the load–displacement curve’s elastic region. This value helped to create an offset line to find the yield point. Yield load refers to the threshold load value that has been applied to prevent plastic deformation, and it was obtained by finding the load value of the yield point. Each load–displacement graph had a peak point called the ultimate point. Ultimate load refers to the maximum load endured, which is found by obtaining the load value of the ultimate point.

### 4.7. Serum Parameters

Serum was removed from the blood samples via centrifugation at 3220× *g* for 8 min, and serum was collected. Total protein (Otto Scientific, OttoBC154), total cholesterol (Otto Scientific, OttoBC135), triglyceride (Otto Scientific, OttoBC135), AST (Otto Scientific, OttoBC127), ALT (Otto Scientific, OttoBC128), and ALP (OttoBC124) were determined with autoanalyzer (Mindray-BS400, Mindray Bio-Medical Electronics Co., Ltd., Shenzhen, China) using commercial kits. Serum IgG levels were analyzed by ELISA with a Randox kit (IG3896). (Randox Laboratories Ltd., Crumlin, UK).

### 4.8. Statistical Analysis

Statistical analyses were carried out using the SPSS program version 22 (IBM SPSS Inc., Chicago, IL, USA). The normality of data distribution was checked using the Kolmogorov–Smirnov test. Two-way ANOVA was used to determine the effects of different *Spirulina platensis* and protein levels on different variables. The significance of mean differences between groups was tested using the Tukey test. The level of significance was taken as *p* < 0.05 [81].

## 5. Conclusions

Reducing the crude protein levels in compound feeds by 10% and supplementing them with 0.1% *Spirulina platensis* had provided higher feed efficiency, healthy intestinal epithelia, higher antioxidant enzyme activities, immunomodulation, hypotriglyceridemic effects, and better meat and bone quality characteristics. Therefore, adding 0.1% *Spirulina platensis* to a low-protein diet could be effective for the poultry industry. More multidisciplinary research is required to determine the criteria for developing potential functional ingredients in broiler nutrition. The bioavailability of the antioxidant compounds in *Spirulina platensis* for poultry should also be investigated.

## Figures and Tables

**Table 1:** Nutrient composition and antioxidant properties of *Spirulina platensis*.

Parameter	Value	Parameter	Value
Dry matter, %	93.90	TAS, mmol Trolox eq/kg	2.36
Crude protein, %	69.73	TOS, µmol H_2_O_2_ eq/kg	23.54
Ether extract, %	3.36	OSI, %	0.997
Crude ash, %	6.18	Total amino acids, g/100 g	
Vitamin E (Alpha-tocopherol), mg/kg	2.8	Ornithine	<0.015
Volatile oil profile, %		Lysine	1.826
3-methyl butanal	1.0	Glycine	2.352
2-methyl butyraldehyde	1.7	Sarcosine	<0.013
Dimethyl disulfide	1.8	Alanine	3.183
1-Hexanal	1.3	Serine	2.191
Benzaldehyde	2.9	Proline	1.856
Isophorone	0.8	Valine	2.589
Cyclopenta©Pyran	1.4	Threonine	2.353
4-Methylacetophenone	1.6	Cystine	<0.02
4H-1,3,5-Dithiazine	2.5	Isoleucine	2.376
Beta-cyclocitral	1.7	Leucine	4.141
Indole	3.6	Hydroxyproline	<0.009
5,6,7,7A-tetrahydro-4,4,7A-trimethyl-2(4H)-benzofuranone	4.3	Asparagine	<0.012
n-Octadecane	2.4	Aspartic acid	4.128
n-Tetradecane	73.1	Glutamic acid	7.035
Fatty acids, % of total fatty acids methyl esters		Methionine	0.371
Myristic acid	3.25	Histidine	0.642
Palmitic acid	43.76	Phenylalanine	2.312
Palmitoleic acid	7.20	Arginine	2.850
Stearic acid	3.81	Citrulline	<0.023
Oleic acid	4.12	Tyrosine	1.950
Linoleic acid	13.76	Tryptophan	<0.046
GLA-gamma-linolenic acid	22.39		
Dihomo-GLA	1.79	Total amino acids	42.15

TAS: Total Antioxidant Status, mmol Trolox equivalent/L; TOS: Total Oxidant Status, µmol H_2_O_2_ equivalent/kg; OSI: Oxidative Stress Index, GLA: Gamma linolenic acid.

**Table 2:** The effect of *Spirulina platensis* supplementation in diets containing normal and low levels of protein on the mean body weight (g) and the mean body weight gain (g) of broilers.

*Spirulina platensis* Level (%)	Protein	Body Weight (g)				Body Weight Gain (g)				
		Day 0	Day 10	Day 24	Day 41	0–10 Day	11–24 Day	0–24 Day	25–41 Day	0–41 Day
0		43.27	268	1099	2774	225	831	1056	1675	2731
0.1		43.55	269	1115	2834	225	846	1071	1719	2790
0.2		43.86	269	1111	2806	225	841	1067	1696	2763
SEM		0.21	1.93	11.60	21.70	0.21	1.88	11.10	11.62	19.02
	Normal	43.61	278	1162	2913	234	884	1118	1751	2870
Protein Level	Low	43.51	260	1054	2696	216	794	1011	1642	2653
SEM		0.18	1.57	9.50	17.70	0.18	1.54	9.10	9.53	15.50
0	Normal	43.47	277	1152	2899	234	875	1109	1747	2856
	Low	43.08	259	1046	2649	216	787	1003	1603	2606
0.1	Normal	43.29	277	1159	2927	234	882	1116	1768	2883
	Low	43.82	260	1070	2741	216	810	1026	1671	2697
0.2	Normal	44.08	279	1175	2914	235	896	1131	1739	2870
	Low	43.64	260	1046	2699	216	787	1003	1652	2656
SEM		0.30	2.72	16.40	30.70	0.30	2.66	15.71	16.43	26.82
*Spirulina platensis* level		0.167	0.918	0.624	0.168	0.979	0.631	0.640	0.273	0.171
Protein level		0.682	<0.001	<0.001	<0.001	˂0.001	˂0.001	˂0.001	˂0.001	˂0.001
*Spirulina platensis* × Protein level		0.211	0.948	0.486	0.586	0.984	0.488	0.503	0.529	0.594

SEM: Standard error of means.

**Table 3:** The effect of *Spirulina platensis* supplementation in diets containing normal and low levels of protein on feed consumption (g), feed conversion ratio (g feed consumption/g body weight gain), and EPEF in broilers by periods.

*Spirulina platensis* Level (%)	Protein	Feed Consumption (g)					Feed Conversion Ratio					EPEF
	Level	0–10 Day	11–24 Day	0–24 Day	25–41 Day	0–41 Day	0–10 Day	11–24 Day	0–24 Day	25–41 Day	0–41 Day	
0		288	1195	1483	2835	4318	1.28	1.44	1.41	1.70 a	1.58 a	428
0.1		289	1201	1490	2832	4322	1.29	1.42	1.39	1.65 b	1.55 b	447
0.2		290	1192	1482	2821	4303	1.29	1.42	1.39	1.67 ab	1.56 b	430
SEM		3.87	12.30	13.40	20.43	26.94	0.02	0.02	0.01	0.01	0.01	7.11
	Normal	297	1219	1516	2859	4375	1.27	1.38	1.36	1.63	1.52	463
	Low	281	1173	1454	2800	4254	1.30	1.48	1.44	1.71	1.60	407
SEM		3.16	9.99	10.91	16.68	21.90	0.01	0.01	0.01	0.01	0.01	5.82
0	Normal	295	1207	1502	2885	4387	1.26	1.38	1.36	1.65	1.54	460
	Low	281	1183	1464	2786	4249	1.30	1.50	1.46	1.74	1.63	396
0.1	Normal	298	1219	1517	2834	4350	1.27	1.38	1.36	1.60	1.51	473
	Low	281	1183	1464	2831	4294	1.30	1.46	1.43	1.70	1.59	420
0.2	Normal	299	1231	1529	2857	4387	1.27	1.37	1.35	1.64	1.53	454
	Low	281	1152	1434	2785	4219	1.30	1.47	1.43	1.69	1.59	405
SEM		5.48	17.40	19.89	18.90	38.00	0.02	0.02	0.02	0.02	0.01	10.10
		*p* value										
*Spirulina platensis* level		0.929	0.861	0.891	0.879	0.862	0.961	0.457	0.558	0.032	0.023	0.143
Protein level		0.001	0.003	<0.001	0.019	0.001	0.130	<0.001	<0.001	<0.001	<0.001	<0.001
*Spirulina platensis* × Protein level		0.933	0.279	0.308	0.245	0.326	0.941	0.537	0.520	0.281	0.346	0.759

a, b: The difference between the mean values with different letters in the same column is statistically significant (*p* < 0.05); SEM: standard error of means; EPEF: European Production Efficiency Factor.

**Table 4:** The effect of *Spirulina platensis* supplementation in diets containing normal and low levels of protein on carcass yield (%) and the relative percentages of organs (g/100 g slaughtering weight).

*Spirulina platensis* Level (%)	Protein Level	Carcass Yield	Liver	Heart	Spleen	Bursa of Fabricius	Gizzard	Abdominal Fat
0		71.52	1.70	0.42	0.11	0.19	1.26	1.38
0.1		71.62	1.70	0.42	0.11	0.20	1.25	1.29
0.2		71.94	1.71	0.41	0.11	0.21	1.25	1.31
SEM		0.233	0.018	0.008	0.005	0.007	0.025	0.052
	Normal	72.01	1.66	0.41	0.10	0.20	1.23	1.12
	Low	71.37	1.75	0.42	0.11	0.20	1.27	1.53
SEM		0.190	0.015	0.006	0.004	0.006	0.020	0.043
0	Normal	71.93	1.65	0.41	0.10	0.19	1.24	1.18
	Low	71.11	1.76	0.43	0.11	0.19	1.28	1.57
0.1	Normal	71.58	1.66	0.42	0.10	0.20	1.23	1.07
	Low	71.66	1.75	0.42	0.11	0.20	1.27	1.51
0.2	Normal	72.53	1.66	0.41	0.11	0.21	1.23	1.09
	Low	71.35	1.76	0.41	0.11	0.20	1.26	1.52
SEM		0.329	0.025	0.011	0.007	0.010	0.035	0.074
		*p* value						
*Spirulina platensis* level		0.413	0.979	0.844	0.636	0.496	0.928	0.485
Protein level		0.020	<0.001	0.538	0.294	0.988	0.223	<0.001
*Spirulina platensis* × Protein level		0.150	0.933	0.490	0.831	0.921	0.987	0.934

SEM: standard error of means.

**Table 5:** The effect of *Spirulina platensis* supplementation in diets containing normal and low levels of protein on some meat characteristics in broilers.

*Spirulina platensis* Level (%)	Protein Level	Color Parameters						Total Phenolic Content (mg GAE/g— Fresh Thigh Meat)	Malondialdehyde (mg/kg— Fresh Thigh Meat)
		Breast Meat			Thigh Meat				
		L*	a*	b*	L*	a*	b*		
0		58.57 b	1.68	5.84	55.56	3.23	5.52 b	1.22	0.38
0.1		60.30 ab	2.01	6.18	55.74	4.02	6.17 ab	1.24	0.35
0.2		60.90 a	2.04	6.73	55.60	4.08	6.64 a	1.28	0.32
SEM		0.538	0.155	0.288	0.542	0.273	0.298	0.025	0.019
	Normal	59.86	1.93	6.23	55.55	3.54	5.75	1.27	0.36
	Low	59.99	1.90	6.27	55.71	4.01	6.47	1.23	0.34
SEM		0.439	0.127	0.235	0.443	0.223	0.244	0.021	0.015
0	Normal	57.36	1.66	5.87	54.55	2.84	5.05	1.25	0.39
	Low	59.78	1.71	5.81	56.57	3.62	5.99	1.20	0.38
0.1	Normal	60.46	2.09	6.21	56.08	3.89	5.76	1.26	0.36
	Low	60.15	1.93	6.16	55.39	4.15	6.56	1.23	0.34
0.2	Normal	61.75	2.03	6.61	56.02	3.91	6.44	1.30	0.33
	Low	60.05	2.05	6.84	55.17	4.25	6.84	1.25	0.31
SEM		0.761	0.219	0.407	0.766	0.387	0.422	0.036	0.026
		*p* value							
*Spirulina platensis* level		0.011	0.211	0.101	0.971	0.059	0.037	0.347	0.084
Protein level		0.827	0.861	0.917	0.795	0.150	0.042	0.155	0.464
*Spirulina platensis* × Protein level		0.031	0.867	0.921	0.122	0.773	0.798	0.969	0.977

L*: lightness, a*: redness, b*: yellowness, a, b: the difference between mean values with different letters in the same column is statistically significant (*p* < 0.05); SEM: standard error of means.

**Table 6:** The effect of *Spirulina platensis* supplementation in diets containing normal and low levels of protein on antioxidant–oxidant status and antioxidant enzyme levels in liver and breast meat of broilers.

		Liver						Breast Meat					
*Spirulina platensis* Level (%)	Protein Level	TAS	TOS	OSI	CAT	SOD	GPx	TAS	TOS	OSI	CAT	SOD	GPx
0		2.69 b	13.77	0.52 a	178 b	164 b	384 b	1.09	0.23	0.02	393	187	458 b
0.1		3.07 a	12.44	0.41 b	204 ab	201 a	439 ab	1.16	0.23	0.02	405	203	542 a
0.2		3.14 a	12.57	0.40 b	220 a	223 a	460 a	1.27	0.22	0.02	415	214	551 a
SEM		0.094	0.558	0.021	9.688	9.450	17.512	0.107	0.023	0.002	18.743	8.469	27.036
	Normal	2.97	13.36	0.46	206	193	435	1.15	0.21	0.02	410	205	519
	Low	2.96	12.49	0.43	196	199	421	1.67	0.24	0.02	399	198	515
SEM		0.077	0.456	0.017	7.910	7.716	14.299	0.034	0.019	0.002	15.304	6.915	22.075
0	Normal	2.72	14.18	0.53	180	164	388	1.05	0.22	0.02	395	188	465
	Low	2.65	13.36	0.51	176	163	380	1.09	0.25	0.02	391	186	451
0.1	Normal	3.04	13.05	0.43	212	202	449	1.10	0.22	0.02	411	206	541
	Low	3.11	11.83	0.38	197	201	430	1.19	0.24	0.02	399	200	544
0.2	Normal	3.16	12.85	0.41	227	214	470	1.13	0.21	0.02	425	220	552
	Low	3.13	12.29	0.39	214	233	541	1.21	0.23	0.02	406	209	549
SEM		0.134	0.790	0.029	13.701	13.364	24.766	0.047	0.032	0.003	26.506	11.977	38.235
		*p* value											
*Spirulina platensis* level		0.003	0.190	˂0.001	0.013	˂0.001	0.011	0.089	0.886	0.578	0.696	0.082	0.037
Protein level		0.942	0.187	0.282	0.361	0.593	0.466	0.081	0.378	0.647	0.601	0.510	0.878
*Spirulina platensis* × Protein level		0.870	0.914	0.804	0.915	0.699	0.972	0.848	0.997	0.995	0.961	0.917	0.976

a, b: means within a column with different letters indicate statistically significant differences (*p* < 0.05). TAS: total antioxidant status, mmol Trolox equivalent/L; TOS: total oxidant status, µmol H_2_O_2_ equivalent/kg; OSI: oxidative stress index; CAT: catalase, U/L; SOD: superoxide dismutase, U/mL; GPx: glutathione peroxidase, U/L; SEM: standard error of mean.

**Table 7:** The effect of *Spirulina platensis* supplementation in diets containing normal and low levels of protein on fatty acids methyl esters in breast meat (g/100 g methyl esters of total fatty acids).

*Spirulina platensis* Level (%)	Protein Level	Myris	Palmit	Palmitol	Stearic	Oleic	Linol	Arach	GLA	Gond	ALA	DGLA	EPA	DHA	SFA	MUFA	PUFA	USA	n6/n3	USA/SFA	PUFA/SFA
0		0.68	17.97	2.56	5.25	30.35	38.05	0.39	0.38	0.35	3.35	0.31	0.22	0.15	24.29	33.26	42.45	75.71	10.45	3.15	1.78
0.1		0.67	17.58	2.60	4.96	30.28	38.65	0.38	0.40	0.35	3.43	0.33	0.23	0.16	23.58	33.23	43.20	76.42	10.34	3.26	1.85
0.2		0.68	17.75	2.82	4.99	30.57	37.95	0.39	0.40	0.35	3.37	0.33	0.23	0.17	23.81	33.74	42.45	76.19	10.25	3.22	1.81
SEM		0.009	0.22	0.11	0.12	0.29	0.45	0.01	0.01	0.01	0.05	0.01	0.01	0.01	0.30	0.37	0.51	0.30	0.08	0.05	0.04
	Normal	0.66	16.45	2.09	4.93	28.47	41.85	0.38	0.38	0.34	3.76	0.31	0.22	0.17	22.41	30.89	46.70	77.59	10.25	3.47	2.09
	Low	0.70	19.09	3.23	5.20	32.33	34.58	0.39	0.40	0.37	3.01	0.34	0.23	0.15	25.37	35.93	38.70	74.63	10.44	2.95	1.53
SEM		0.007	0.18	0.09	0.10	0.24	0.37	0.01	0.01	0.01	0.04	0.01	0.01	0.01	0.24	0.31	0.42	0.24	0.07	0.04	0.03
0	Normal	0.67	16.31	1.78	5.10	27.96	42.61	0.39	0.37	0.34	3.82	0.30	0.21	0.17	22.46	30.06	47.48	77.54	10.31	3.46	2.12
	Low	0.70	19.64	3.34	5.40	32.75	33.49	0.38	0.39	0.37	2.89	0.32	0.22	0.13	26.12	36.45	37.43	73.88	10.59	2.84	1.44
0.1	Normal	0.65	16.51	2.28	4.80	28.82	41.41	0.37	0.39	0.33	3.74	0.31	0.22	0.18	22.33	31.43	46.25	77.67	10.19	3.48	2.07
	Low	0.70	18.64	2.93	5.11	31.73	35.88	0.38	0.42	0.37	3.12	0.34	0.23	0.15	24.83	35.03	40.15	75.17	10.49	3.04	1.62
0.2	Normal	0.65	16.51	2.22	4.89	28.63	41.54	0.39	0.39	0.34	3.72	0.32	0.22	0.18	22.45	31.19	46.36	77.55	10.26	3.47	2.07
	Low	0.70	18.98	3.41	5.10	32.52	34.36	0.40	0.40	0.37	3.02	0.34	0.24	0.17	25.17	36.30	38.54	74.84	10.25	2.98	1.54
SEM		0.01	0.32	0.16	0.17	0.41	0.64	0.01	0.02	0.01	0.07	0.01	0.01	0.01	0.42	0.52	0.72	0.42	0.12	0.07	0.06
		*p* value																			
*Spirulina platensis* level		0.652	0.473	0.238	0.210	0.755	0.504	0.399	0.381	0.787	0.516	0.075	0.308	0.075	0.246	0.549	0.496	0.246	0.277	0.337	0.509
Protein level		<0.001	<0.001	<0.001	0.065	<0.001	<0.001	0.648	0.140	<0.001	<0.001	0.003	0.231	0.012	<0.001	<0.001	<0.001	<0.001	0.060	<0.001	<0.001
*Spirulina platensis* × Protein level		0.820	0.173	0.026	0.944	0.094	0.029	0.825	0.704	0.992	0.085	0.933	0.952	0.269	0.364	0.041	0.034	0.364	0.341	0.468	0.145

Myris: myristic, Palmit: palmitic, Palmitol: palmitoleic, Linol: linoleic, Arach: Arachidic, GLA: gamma-linolenic acid, Gond: gondoic acid (11-Eicosenoic acid), ALA: alpha-linolenic acid, DGLA: dihomogamma-linolenic acid, EPA: eicosapentaenoic acid, DHA: docosahexaenoic acid, SFA: saturated fatty acids, MUFA: mono-unsaturated fatty acids, PUFA: polyunsaturated fatty acids, USA: unsaturated fatty acids, n6/n3: the ratio of omega 6/omega 3 fatty acids; SEM: standard error of mean.

**Table 8:** The effect of *Spirulina platensis* supplementation in diets containing normal and low levels of protein on intestinal histomorphology.

*Spirulina platensis* Level (%)	Protein Level	Duodenum			Jejunum			Ileum		
		VH (µm)	CD (µm)	VH/CD	VH (µm)	CD (µm)	VH/CD	VH (µm)	CD (µm)	VH/CD
0		1560 b	80.09 b	22.76 a	1012 b	89.08	11.97 b	780 b	92.14	8.66 b
0.1		1682 a	88.33 a	20.02 b	1111 a	89.13	12.89 a	841 a	91.83	9.47 a
0.2		1693 a	89.00 a	19.65 b	1123 a	89.57	12.97 a	845 a	91.94	9.39 a
SEM		14.380	1.209	0.401	8.460	0.974	0.175	10.081	0.903	0.137
	Normal	1718	89.35	20.01	1104	92.06	12.37	879	92.26	9.82
	Low	1572	82.27	21.62	1059	85.56	12.84	765	91.69	8.53
SEM		11.741	0.987	0.327	6.907	0.795	0.143	8.231	0.737	0.112
0	Normal	1590	88.88	18.41	1095	98.02	11.03	867	92.21	9.72
	Low	1530	71.31	27.10	929	80.14	12.00	692	92.05	7.60
0.1	Normal	1777	89.46	20.89	1105	90.03	12.68	879	92.27	9.87
	Low	1586	87.21	19.15	1117	88.24	13.10	803	91.39	9.07
0.2	Normal	1787	89.72	20.71	1114	90.83	12.50	892	92.26	9.86
	Low	1599	88.28	18.59	1132	88.30	13.44	799	91.62	8.91
SEM		20.337	1.709	0.567	11.964	1.377	0.247	14.257	1.277	0.194
		*p* value								
*Spirulina platensis* level		<0.001	<0.001	<0.001	<0.001	0.928	<0.001	<0.001	0.971	<0.001
Protein level		<0.001	<0.001	0.001	<0.001	<0.001	0.023	<0.001	0.590	<0.001
*Spirulina platensis* × Protein level		0.002	<0.001	<0.001	<0.001	<0.001	0.216	0.002	0.961	0.002

a, b: means within a column with different letters indicate statistically significant differences (*p* < 0.05). SEM: standard error of means; VH: villus height, CD: crypt depth.

**Table 9:** The effect of *Spirulina platensis* supplementation in diets containing normal and low levels of protein on tibia and femur properties.

		Tibia				Femur			
*Spirulina platensis* Level (%)	Protein level	UL (N)	Stiffness (N/mm)	YL (N)	DYL (mm)	UL (N)	Stiffness (N/mm)	YL (N)	DYL (mm)
0		413	379	346	1.15 b	324 b	306	297	1.50
0.1		416	364	354	1.37 a	338 ab	314	293	1.65
0.2		423	364	359	1.49 a	359 a	322	308	1.70
SEM		8.819	10.387	9.125	0.044	7.557	7.716	5.877	0.079
	Normal	464	340	369	1.16	349	318	311	1.58
	Low	371	398	337	1.51	332	310	288	1.65
SEM		7.201	8.481	7.450	0.036	6.170	6.300	4.799	0.065
0	Normal	454	345	353	1.04	341	311	313	1.54
	Low	372	413	340	1.26	308	302	281	1.45
0.1	Normal	462	335	369	1.21	341	314	302	1.59
	Low	371	392	338	1.53	335	314	284	1.70
0.2	Normal	476	340	385	1.23	365	328	318	1.60
	Low	370	388	334	1.74	353	315	299	1.79
SEM		12.472	14.689	12.904	0.063	10.687	10.912	8.311	0.112
		*p* value							
*Spirulina platensis* level		0.722	0.482	0.608	<0.001	0.007	0.381	0.174	0.188
Protein level		<0.001	<0.001	0.003	<0.001	0.051	0.415	0.001	0.437
*Spirulina platensis* × Protein level		0.641	0.800	0.349	0.069	0.414	0.847	0.628	0.440

a, b: means within a column with different letters indicate statistically significant differences (*p* < 0.05). UL: ultimate load, YL: yield load, DYL: displacement at yield load; SEM: standard error of means.

**Table 10:** The effect of *Spirulina platensis* supplementation in diets containing normal and low levels of protein on blood serum biochemical parameters and IgG levels of broilers.

*Spirulina platensis* Level (%)	Protein Level	Total Protein (g/dL)	Trigly (mg/dL)	Total Cholest (mg/dL)	ALP (U/L)	AST (U/L)	ALT (U/L)	IgG (mg/mL)
0		2.15	50.25 b	119	4158	291	7.81	1.87 b
0.1		2.16	40.50 a	112	4187	282	6.50	2.95 a
0.2		2.22	39.00 a	112	4170	283	6.94	3.04 a
SEM		0.158	3.394	5.938	293	18.494	0.704	0.194
	Normal	2.23	36.63	109	4172	304	6.79	3.00
	Low	2.12	49.88	119	4172	267	7.38	2.34
SEM		0.129	2.771	4.848	239	15.100	0.575	0.158
0	Normal	2.21	44.50	115	4167	309	7.88	1.93
	Low	2.09	56.00	123	4149	274	7.75	1.81
0.1	Normal	2.21	33.00	104	4180	294	6.25	3.48
	Low	2.12	48.00	119	4193	271	6.75	2.43
0.2	Normal	2.28	32.38	107	4167	310	6.25	3.60
	Low	2.16	45.63	117	4173	257	7.63	2.47
SEM		0.224	4.800	8.397	414	26.154	0.995	0.274
		*p* value						
*Spirulina platensis* level		0.949	0.049	0.616	0.998	0.933	0.413	˂0.001
Protein level		0.538	0.002	0.141	1.000	0.091	0.477	0.001
*Spirulina platensis* × Protein level		0.996	0.936	0.899	0.999	0.848	0.752	0.137

a, b: means within a column with different letters indicate statistically significant differences (*p* < 0.05). ALP: alkaline phosphatase; AST: aspartate aminotransferase; ALT: alanine aminotransferase, Trigly: triglyceride, Cholest: cholesterol, IgG: immunoglobulin G; SEM: standard error of means.

**Table 11:** Ingredients and chemical composition of the diets (as-fed basis).

	Normal Protein Group			Low-Protein Group		
Ingredients (%)	Starter (0–10 d)	Grower (10–24 d)	Finisher (24–41 d)	Starter (0–10 d)	Grower (10–24 d)	Finisher (24–41 d)
Maize	50.32	53.35	57.55	57.19	59.74	63.35
Soybean meal	30.00	21.20	16.20	27.80	18.96	14.12
Fullfat soya	14.97	20.90	21.00	10.30	16.75	17.28
Soy oil	0	0	1.00	0	0	1.00
Monocalcium phosphate	2.00	2.00	1.90	2.00	2.00	1.90
Limestone	1.50	1.40	1.30	1.50	1.40	1.30
Sodium bicarbonate	0.10	0.10	0.10	0.10	0.10	0.10
Salt	0.25	0.25	0.25	0.25	0.25	0.25
DL-Methionine	0.30	0.30	0.25	0.30	0.30	0.25
L-Lysine	0.20	0.20	0.15	0.20	0.20	0.15
Threonine	0.10	0.10	0.10	0.10	0.10	0.10
Vitamin premix ^1^	0.10	0.10	0.10	0.10	0.10	0.10
Mineral premix ^2^	0.10	0.10	0.10	0.10	0.10	0.10
Anticoccidial	0.06	0	0	0.06	0	0
**Diet Analysis**						
Dry matter, %	92.23	92.33	92.12	92.06	92.25	92.32
Crude protein, %	22.98	21.44	19.49	20.82	19.33	17.50
Ether extract, %	6.05	6.80	7.98	5.88	6.24	7.47
Crude fibre, %	3.90	3.78	3.70	3.73	3.75	3.59
Crude ash, %	6.15	5.50	6.06	6.02	6.10	5.47
Calcium, %	1.19	0.98	1.08	1.08	1.16	0.99
Total phosphorus, %	0.82	0.77	0.80	0.81	0.84	0.79
Metabolizable energy, kcal/kg	3010	3120	3210	3004	3100	3215
ME (kcal)/CP (g)	13.04	16.41	16.02	14.42	14.49	18.23

^1^ Vitamin Premix (per 1 kg): contains 11,000,000 IU of vitamin A, 3,500,000 IU of vitamin D3, 100 g of vitamin E, 3 g of vitamin K3, 3 g of vitamin B1, 6 g of vitamin B2, 15 g of calcium D-pantothenate, 1 g of vitamin B6, 35 g of niacin, 1.5 g of folic acid, 200 mg of biotin, and 20 mg of vitamin B12. ^2^ Mineral Premix (per 1 kg): contains 30 g of copper, 120 g of manganese, 110 g of zinc, 2 g of iodine, 300 mg of selenium, and 50 g of iron.

## Data Availability

The data that support the findings of this study are available from the corresponding author upon reasonable request.
